# Lower respiratory tract microbiota characteristics in patients with *Pseudomonas aeruginosa* pneumonia during antibiotic therapy

**DOI:** 10.3389/fcimb.2026.1777821

**Published:** 2026-05-13

**Authors:** Yongchao Jiang, Rong Wang, Xin Qin, Liang Shen, Hui Xing, Fan Ye, Lijuan Yin, Yang Yang, Chunhua Wang, Tangjie Xu, Mengjun Tang

**Affiliations:** 1 Department of Central Laboratory, Xiangyang Central Hospital, Affiliated Hospital of Hubei University of Arts and Science, Xiangyang, Hubei, China; 2 Department of Pediatrics, Xiangyang Central Hospital, Affiliated Hospital of Hubei University of Arts and Science, Clinical Research Center for Pediatric Diseases and Rare Diseases, Xiangyang, Hubei, China; 3 School of Basic Medicine, Hubei University of Arts and Science, Xiangyang, Hubei, China; 4 College of Biotechnology, Tianjin University of Science & Technology, Tianjin, China; 5 Shenzhen Key Laboratory of Pathogen and Immunity, National Clinical Research Center for Infectious Disease, State Key Discipline of Infectious Disease, Shenzhen Third People’s Hospital, Second Hospital Affiliated to Southern University of Science and Technology, Shenzhen, China; 6 Department of Gynecology and Obstetrics, First Affiliated Hospital of Guangzhou Medical University, Guangzhou, Guangdong, China

**Keywords:** antibiotic therapy, elderly patients, lower respiratory tract microbiota, microbial biomarkers, *Pseudomonas aeruginosa* pneumonia

## Abstract

**Introduction:**

*Pseudomonas aeruginosa* (Pae) is a major cause of bacterial pneumonia in older adults, yet the composition of the lower respiratory tract (LRT) microbiota in patients with *P. aeruginosa pneumonia* (PAP), and its potential prognostic relevance, remain poorly defined.

**Methods:**

Here, we performed 16S rRNA gene sequencing of bronchoalveolar lavage fluid collected from older adults with PAP at hospital admission and discharge, yielding 42 paired samples. We characterized longitudinal microbiota dynamics, identified candidate microbial biomarkers using LEfSe and random forest analyses, inferred functional profiles with Tax4Fun2, and compared microbiota-associated changes following meropenem-based therapy (n = 12) or levofloxacin-based therapy (n = 9).

**Results:**

PAP was associated with marked shifts in LRT microbial community structure, with post-treatment samples showing reduced *Pseudomonas* abundance and increased relative abundances of *Rothia, Streptococcus* and *Porphyromonas* (*p* < 0.05). Random forest modeling identified *P. aeruginosa, Streptococcus pneumoniae*, and *Prevotella melaninogenica* as potential diagnostic biomarkers (AUC = 0.93). Functional prediction further suggested a reduction in biofilm-formation-related pathways after treatment (*p* < 0.01). Notably, Pseudomonas abundance correlated positively with white blood cell count and C-reactive protein, but inversely with lymphocyte and platelet counts, supporting the potential value of these clinical indices in prognostic assessment. In this cohort, meropenem-based therapy, compared with levofloxacin -based therapy, was associated with enrichment of several potentially pathogenic anaerobic genera, including *Porphyromonas, Campylobacter, Neisseria* and *Prevotella*.

**Discussion:**

Collectively, these findings define the LRT microbiota landscape in elderly patients with PAP, nominate candidate microbial biomarkers, and indicate that antibiotic regimen is associated with distinct post-treatment microbial community structures, with potential implications for therapeutic decision-making.

## Introduction

Respiratory tract infections represent one of the leading causes of global mortality (GBD 2017 Influenza Collaborators, 2019). Previous studies have established a link between dysbiosis of the lower respiratory tract (LRT) microbiota and various LRT infections (Dickson et al., 2020; Thibeault et al., 2021; Zhu et al., 2023). Pae, commonly known as green pus bacillus, is a representative species of the non-fermenting bacteria family *Pseudomonadaceae*, belonging to the Gram-negative bacilli. Pae is a major bacterial pathogen in most cases of infectious pneumonia in adults and ranks as one of the common opportunistic pathogens in clinical settings (Ihssane et al., 2023; Moradali et al., 2017). Additionally, Pae exhibits strong survival capabilities, readily forming biofilms, and can colonize the lungs of cystic fibrosis (CF) patients, leading to difficult-to-eradicate chronic infections (Beaudoin et al., 2018; Sando et al., 2021). Pae tends to infect immunocompromised patients, such as the elderly, those with metabolic disorders, hematologic malignancies, or postoperative patients (Sommer et al., 2024). Due to the weakened immune defenses, altered drug metabolism, and impaired immune function in these populations, this can precipitate or exacerbate organ failure, worsening diseases, leading to high morbidity and mortality rates (Whelan et al., 2014). Despite antibiotics serve as the primary treatment for PAP, the intrinsic and acquired resistance of Pae to antibiotics, along with its intricately complex intracellular regulatory networks, present significant challenges in the management of PAP (Hu et al., 2022). Furthermore, prolonged antibiotic use can escalate the risk of multidrug-resistant Pae in patients. This escalating antimicrobial resistance is of particular concern in older adults, in whom PAP may evolve into persistent or recurrent infection that responds poorly to antimicrobial therapy, thereby increasing the risks of sepsis, respiratory failure, and adverse clinical outcomes (Pang et al., 2019; Tenover et al., 2022).

In recent years, the upgrading and widespread use of various anti-microbial agents has impacted the distribution of respiratory tract microbiota and pathogens (Bassetti and Righi, 2015; Javanmardi et al., 2019). The choice of medication for PAP would directly affect patient prognosis, including cure rates and mortality rates (Hespanhol and Bárbara, 2020). Reducing pulmonary complications and alleviating adverse effects of empirical antibiotic therapy remain significant challenges for clinicians and microbiologists (Luu and Muhsin, 2022). Recent studies suggest that the composition of LRT microbial communities may play a crucial role in the prevention and treatment of PAP (Fangous et al., 2021; Khailova et al., 2013; Shoaib et al., 2019). *Khailova* et al. demonstrated that *Lactobacillus rhamnosus* can reduce lung Pae loads and increase the survival rates in PAP mice (Khailova et al., 2013). Additionally, patients pre-treated with probiotics showed a reduced likelihood of LRT Pae colonization, indicating that microbiota regulation could be a viable strategy against Pae infection (Fangous et al., 2021). However, there is scarce literature on which bacteria can influence the progression of PAP. To enhance the understanding of the diversity of LRT microbiota in PAP patients, our study aims to examine the dynamic characteristics of the LRT microbiome and identify potential microbial biomarkers in bronchoalveolar lavage fluid (BALF) samples using 16S rRNA gene amplicon sequencing. Furthermore, we explored the correlation between microbial composition and clinical characteristics and prognosis of PAP patients. This study will provide valuable insights into the distribution of LRT microbiota during the infection process in PAP patients, thereby offering guidance for clinical treatment strategies for such patients.

## Materials and methods

### Participants

21 adult patients (> 60 years old) were recruited from the department of respiratory and critical care of Xiangyang Central Hospital (Hubei, China) for intravenous antibiotic treatment due to acute infectious pulmonary exacerbation from March 2023 to March 2024. Antibiotic selection was based on antimicrobial susceptibility testing (AST): meropenem for carbapenem−susceptible strains and levofloxacin for fluoroquinolone−susceptible strains. Inclusion criteria of PAP patients included: (1) no mechanical ventilation, (2) satisfied two of the following: body temperature > 38 °C or < 36 °C, leukopenia or leukocytosis, or purulent secretions, (3) new or progressive chest infiltrates, for patients with underlying pulmonary or cardiac disease, two serial chest radiographs were required for assessment, (4) endotracheal aspiration cultured Pae at least ++ growth using semi-quantitative measurements (Kalil et al., 2016). Exclusion criteria included: age < 60 years, pregnant woman, no interpretable chest X-ray was obtained, diagnosed as a non-immunocompromised disease or preexisting lung disease (e.g. COPD, CF or non-CF bronchiectasis). Written informed consents were obtained from all study subjects or their lineal consanguinities prior to enrollment. Clinical data collection was performed at the hospital admission and terminated following study with drawal, discharge, or death.

The patient’s clinical characteristics are presented in **Table 1**. Written informed consent was secured from all participants or their immediate family members prior to enrollment. All protocols in this study were approved by the Ethics Committee of Xiangyang Central Hospital in accordance with the declaration of Helsinki (ethics number: 2023-016-029).

**Table 1:** The characteristics of elderly PAP patients in admission and discharge groups.

Demographics	Cluster (n=21)		
Sex			
Male	12		
Female	9		
Age, y	67 (55-72)		
BMl, kg/m2	23.45 ± 4.12		
Smoker	1		
Drinker	2		
Chronic coexisting disease			
Chronic bronchitis	0		
Diabetes	2		
Hypertension	2		
Characteristics	Admission group	Discharge group	P value
WBC (10^9^)	12.87	6.54	< 0.001
CRP (mg/L)	83.9	16.7	< 0.001
PCT(%)	0.22	0.25	0.233
LYMPH (10^9^)	0.79	1.21	0.021
PLT (10^9^)	84	245	< 0.001
HGB(g/L)	107	114	0.112
RBC (10^12^)	3.42	3.29	0.151
HCT(%)	31.5	32.1	0.185

CRP, C-reactive protein; HCT, hematocrit; HGB, hemoglobin; LYMPH, lymphocyte; PLT, platelet; PCT, procalcitonin; RBC, red blood cell; WBC, white blood cell.

### BALF collection

The BALF procedure was carried out with reference to a standard safety protocol (Miao et al., 2022). For each of the 21 enrolled patients, two BALF samples were obtained: one within 24 hours of hospital admission and the other at discharge. Sampling targeted either the right middle lobe or the left upper lobe of the lung. After the bronchoscope reaches a wedge position, 50 mL of normal saline were instilled. This wedged position was maintained and normal saline was suctioned. The collected samples were immediately transferred to separate sterile 15ml centrifuge tubes (Life Technologies, Mulgrave, VIC, Australia) and stored at -80 °C after volume documentation.

### DNA extraction and sequencing

A total of 42 bronchoalveolar lavage fluid (BALF) samples, collected from 21 patients at two time points, were subjected to 16S rRNA gene amplicon sequencing and downstream bioinformatic analyses. Microbial DNA was extracted and purified utilizing the QIAamp DNA Microbiome Kit (Qiagen, Hilden, Germany). Subsequently, the extracted microbial DNA served as the template for sequencing library construction, facilitated by the TruSeq^®^ DNA PCR-Free Sample Preparation Kit (Illumina, San Diego, CA, USA).

The library was purified and its quality was evaluated using agarose gel electrophoresis. Following a series of dilutions, mixings, and denaturations, the sample was spiked with PhiX as directed by the NovaSeq System Denature and Dilute Libraries Guide. Sequencing operations employed an Illumina NovaSeq system (2 × 150 bp paired-end reads), utilizing the high-output reagent kit for 150 cycles, and were conducted in accordance with the NovaSeq Local Run Manager Software Guide. DNA extraction and sequencing were performed by CapitalBio Corporation (Beijing, China). Amplification of the 16S rRNA gene was accomplished using the primers 338F (5′-barcode-ACTCCTACGGGAGGCAGCAG-3′) and 806R (5′-GGACTACHVGGGTWTC TAAT-3′), targeting the V4 region of the 16S rRNA gene.

### Data processing

High-quality filtering and trimming of the raw FASTQ files were performed using USEARCH 8.0, adhering to the following criteria: (1) exact index matching, (2) only sequences with > 50 bp overlaps were assembled according to their overlap sequence, (3) merged sequences > 400 bp, and (4) read pairs were merged using FLASH (v1.2.11) with strict quality control: minimum overlap 30 bp, maximum mismatch rate in overlap region < 0.01. Subsequently, the filtered reads were assembled into contigs, which were then used for read mapping to calculate the read count per contig. Unassembled reads were discarded from the analysis. Chimeras were identified and removed using UCHIME against the SILVA database (Edgar et al., 2011). Operational taxonomic units (OTUs) were clustered at a 97% similarity threshold utilizing UPARSE version 7.1 (http://www.drive5.com/uparse/), following the removal of chimeric sequences.

Taxonomic classification for OTUs were annotated using the Ribosomal Database Project (RDP) classifier (https://sourceforge.net/projects/rdp-classifier/) with a confidence threshold of 70%, and the resulting data informed subsequent analyses (Quast et al., 2013). The raw sequencing data have been uploaded in the NCBI GenBank Sequence Read Archive database (accession number: PRJNA1103868).

### Bioinformatics analysis

For downstream analysis, all assigned reads were subjected to alpha and beta diversity assessments using the Quantitative Insights into Microbial Ecology (QIIME) package implemented in R version 3.2.1 (Fujimura et al., 2016). Alpha diversity measures were compared using the Wilcoxon signed-rank test, while beta diversity was assessed via non-metric multidimensional scaling.

The sequencing depth of the BALF sample was evaluated by generating rarefaction curve (RC) and rank-abundance curve for each sample. Compositional dissimilarities between samples were quantified using weighted pair-group method with arithmetic means (UPGMA), visualized as principal coordinate analysis (PCoA) plots. LEfSe was applied to identify differentially abundant OTUs between admission and discharge groups in elderly PAP patients (Segata et al., 2011). Functional enrichment analysis of novel genes was conducted using the Gene Ontology (GO) and Kyoto Encyclopedia of Genes and Genomes (KEGG) databases based on Tax4Fun2 (Kanehisa, 2019; Kanehisa and Goto, 2000; Kanehisa et al., 2025), with the aid of the ‘clusterProfiler’ package in R version 4.2.1 (Aßhauer et al., 2015).

### Random forest model construction

A Random Forest classifier was trained on standardized microbial abundance data to discriminate PAP patients before and after antibiotic therapy. Key hyperparameters were optimized via 10-fold cross-validation (training set: 70% samples) by maximizing AUC-ROC. Final model performance was evaluated on an independent test set (30% samples) reporting AUC-ROC, sensitivity, specificity, and feature importance for key discriminative taxa.

### Statistical analysis

The correlation between admission and discharge groups were analyzed using SPSS version 23 (Armonk, New York, USA). Continuous variables were presented as the mean ± standard deviation for normally distributed data and median for non-normally distributed data. Spearman correlations between microbial taxa and clinical variables were computed using SciPy (v1.10.0). Resulting p-values were adjusted for false discovery rate (FDR) using the Benjamini-Hochberg procedure, with *p* < 0.05 considered significant. Figures were generated using GraphPad Prism version 6.0.

## Results

### The demographic and clinical characteristics of PAP patients

As shown in **Table 1**, a total of 21 PAP patients were recruited in this study, comprising 12 males and 9 females, with an average age of 67 years old. Of them, one patient had a history of smoking and other two had a history of alcohol consumption. Concerning chronic diseases, two patients had diabetes, and two had hypertension. Comparing admission to discharge, patients exhibited a significant decrease in serum WBC and CRP concentration, along with a notable increase in LYM and PLT counts (*p* < 0.05). However, other indicators such as procalcitonin (PCT), hemoglobin (HGB), red blood cell (RBC) count, and hematocrit (HCT) did not show significant differences between admission and discharge (*p >* 0.05). These markers were descriptively assessed, but only WBC, CRP, LYM, and PLT showed significant within-cohort changes.

### LRT microbiota of PAP patients

**Figure 1:**
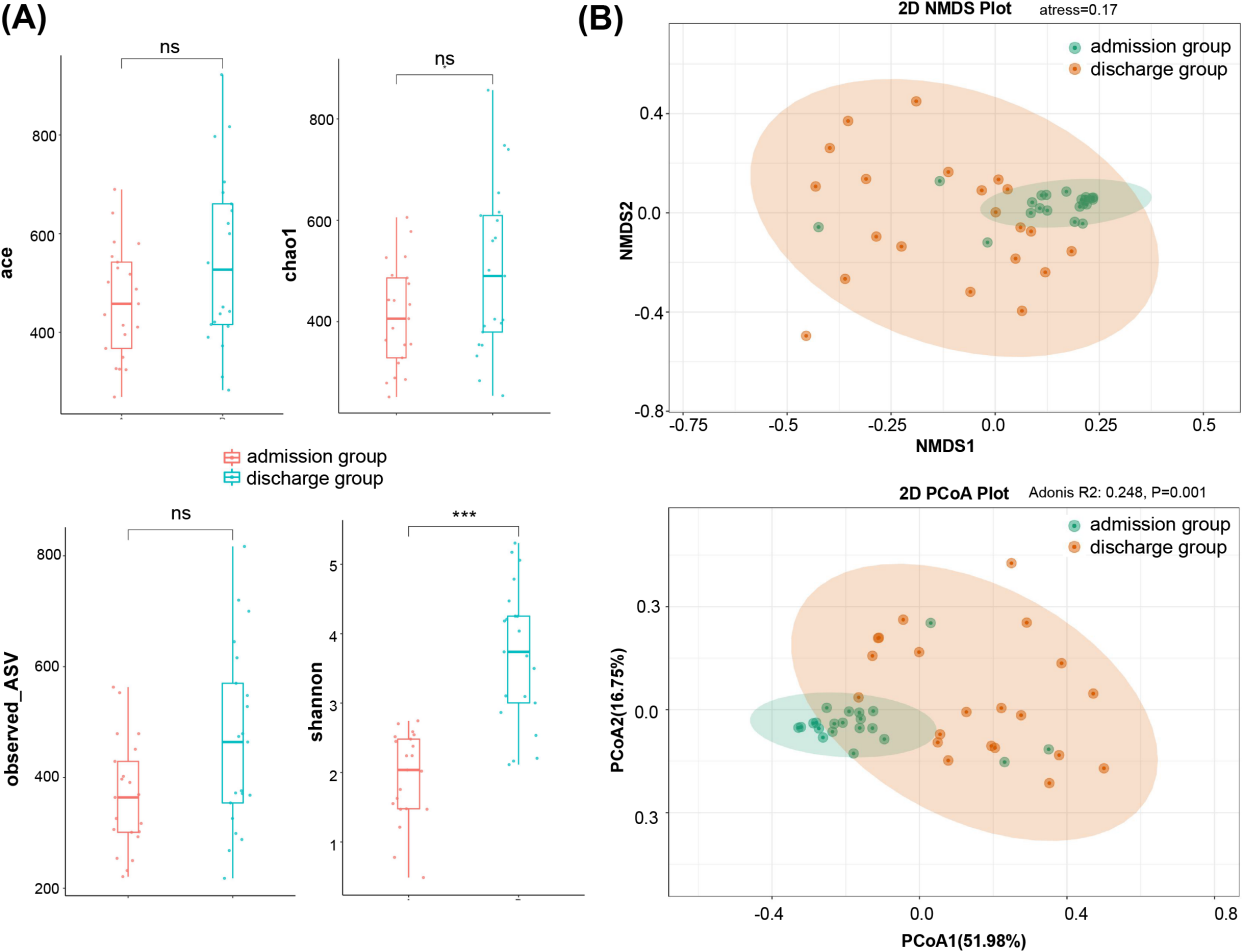
Diversity analysis for microbiota in BALF. **(A)** Alpha diversity analysis showed significant differences in abundance-based coverage estimator (ACE), taxonomy-based richness (Chao1), observed OTUs and Shannon index, **(B)** Beta diversity analysis by non-metric multidimensional scaling (NMDS, up panel) and principal co-ordinates analysis (PCoA, down panel) suggesting the community structure of microbiota. Each dot represents one sample.

To investigate how antibiotic treatment influenced lower respiratory tract (LRT) microbial communities during hospitalization, we performed 16S rRNA gene amplicon sequencing on 42 BALF samples collected longitudinally from 21 patients at admission and discharge. Total 3,716,744 valid reads were obtained from the samples (ranging from 73,016 to 104,417 reads per sample), which could be classified into 16 phyla, including 354 genera (**Supplementary Table 1**). The average coverage was 99.76 ± 0.12%, and dilution curves and abundance curves showed clear asymptotes (**Supplementary Figure 1**), indicating sufficient sequencing depth for microbial community analysis of BALF samples. Alpha diversity, represented by ACE (*p* = 0.13), Chao 1 (*p* = 0.09), and observed OTUs (*p* = 0.06), showed no significant differences between the admission and discharge groups (**Figure 1A**). However, the Shannon index of the admission group was significantly lower than that of the discharge group (*p* < 0.001), indicating significant variation in alpha diversity between the two groups. Phylogenetic analysis revealed significant differences in the composition of LRT microbial communities between the admission and discharge groups. Principal coordinate analysis (PCoA) plots based on weighted UniFrac distances (**Figure 1B**, **Supplementary Figure 2**) revealed two distinct clusters (R^2^ = 0.38, *p<* 0.01).

### Effect of antibiotic treatment on the abundance of microbial taxa in LRT infection patients

**Figure 2:**
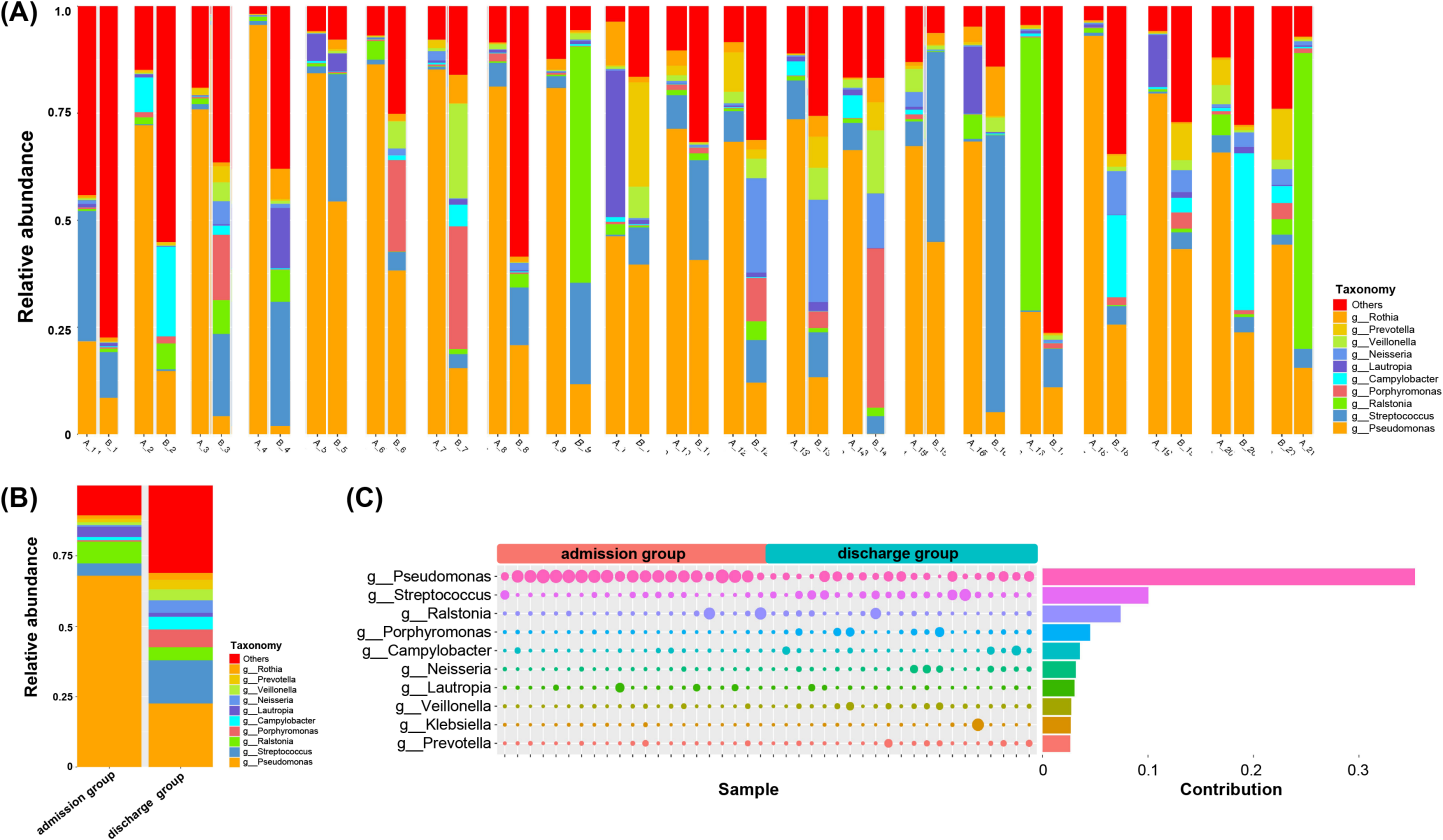
The characteristics of BALF microbiota at genus level. **(A)** Dynamic variation of LRT microbiota in PAP patients at the time of initial and 8–10 day sample collection. A-admission group, B-discharge group, **(B)** Analysis of simper contribution of species between the two groups. The size corresponds to their relative contribution in the respective samples. Student t-test was used to evaluate significant differences between the groups. *p < 0.05, **(C)** Relative abundance of microbiota in admission group (initial sample) and discharge group (sample from days 8-10).

To investigate the impact of antibiotic treatment on the lung microbial communities in PAP patients, beta diversity analysis was conducted. We observed fluctuations in the major species in samples collected upon admission and discharge, particularly an increase in microbial diversity at the genus level (**Figure 2A**). Subsequently, we compared the relative abundance of the ten most abundant genera (on average) across all samples in both the admission and discharge groups, including *Rothia, Prevotella, Veillonella, Neisseria, Lautropia, Campylobacter, Porphyromonas, Ralstonia, Streptococcus, and Pseudomonas* (**Figure 2B**). Comparing to patients upon admission, the relative abundance of *Pseudomonas* in the LRT of discharged patients was significantly reduced (*p* < 0.001). Moreover, the relative abundance of *Rothia* (*p* = 0.013), *Prevotella* (*p* = 0.009), *Streptococcus* (*p* = 0.001), *Campylobacter* (*p* < 0.001)*, Porphyromonas* (*p* < 0.003), *Neisseria* (*p* = 0.006) and *Veillonella* (*p* = 0.019) in the LRT of discharged patients was significantly increased. To further confirm these findings, we constructed the phylogenetic tree and calculated the contributions of distinct genera between the two groups, which were consistent with the above conclusions (**Figure 2C**, **Supplementary Figure 3**).

### LEfSe analysis identifies the dysregulated microbial communities in PAP patients

**Figure 3:**
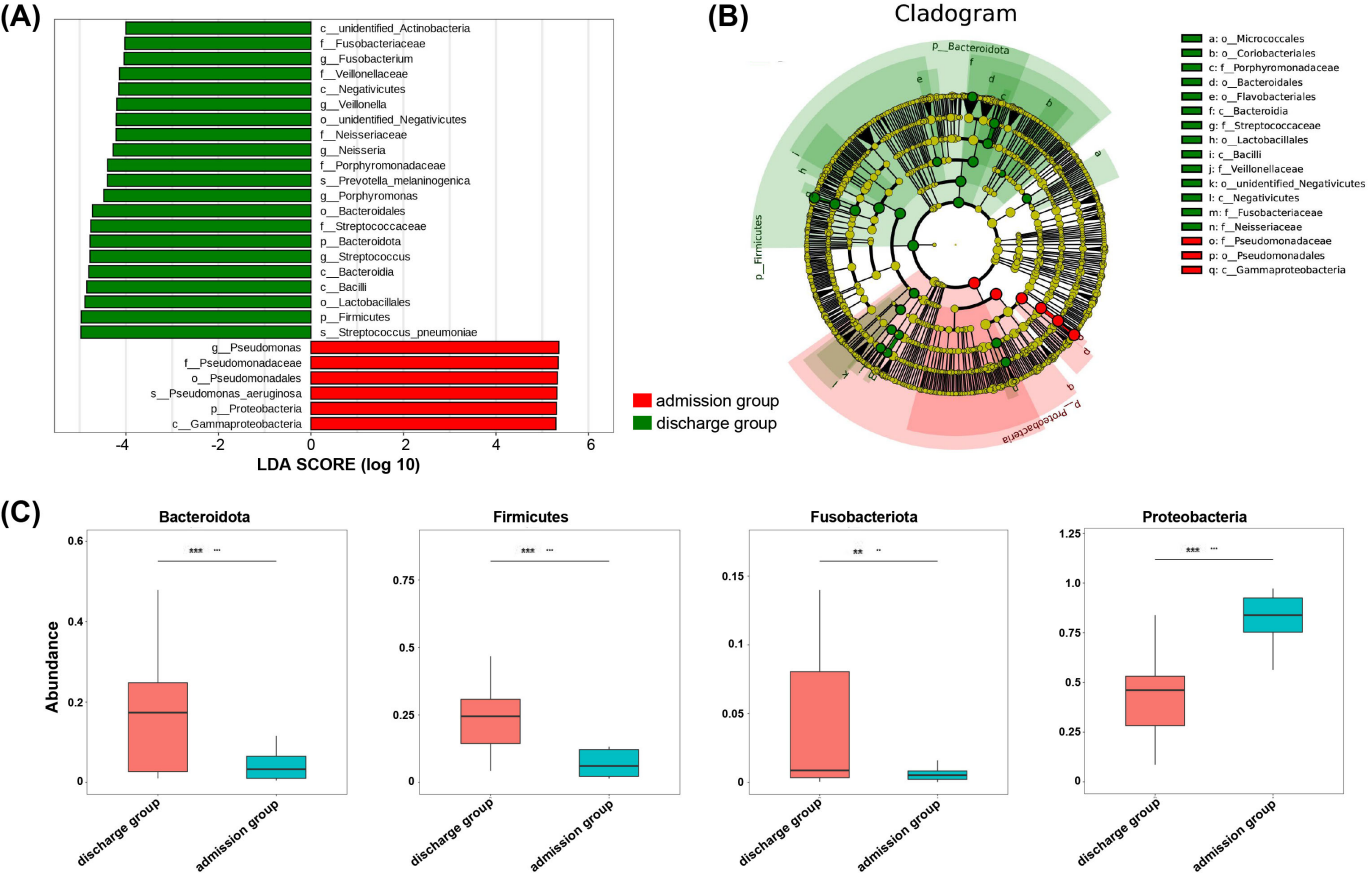
The difference of BALF microbiota composition between discharge group and admission group of elderly PAP patients based on LEfSe version 1.0 (https://huttenhower.sph.harvard.edu/lefse/). **(A)** The LDA scores of taxa presented the difference in microbiome composition between two groups, LDA score greater than 4 is a biomarker with statistical differences between groups, **(B)** Taxonomic cladogram by LEfSe analysis showed the changes of microbiome, **(C)** Analysis of significant difference of microbiota between two groups. Student t-test was used to evaluate significant difference. *p < 0.05.

LEfSe analysis was used to identify potential indicators of disease progression and treatment response in the LRT of PAP patients from admission to discharge. This analysis revealed that 27 taxonomic groups exhibited significant differences in abundance before and after antibiotic treatment, with *Pseudomonas* yielded the highest linear discriminant analysis (LDA) score, indicating its strong influence on microbial module groups (**Figure 3A**). Additionally, several microbial taxa, such as *Proteobacteria*, *Firmicutes*, and *Bacteroidetes*, appeared as potential biomarkers (**Figure 3B**). Notably, analysis of significant differences identified several dysregulated taxonomic groups, the decrease in *Proteobacteria* and the increase in *Firmicutes*, *Fusobacteriota* and *Bacteroidetes* after antibiotic treatment may reflect the recovery of the ecosystem following the inhibition of *Pseudomonas* (**Figure 3C**).

### Potential pathogenic microbes screening in PAP patients

**Figure 4:**
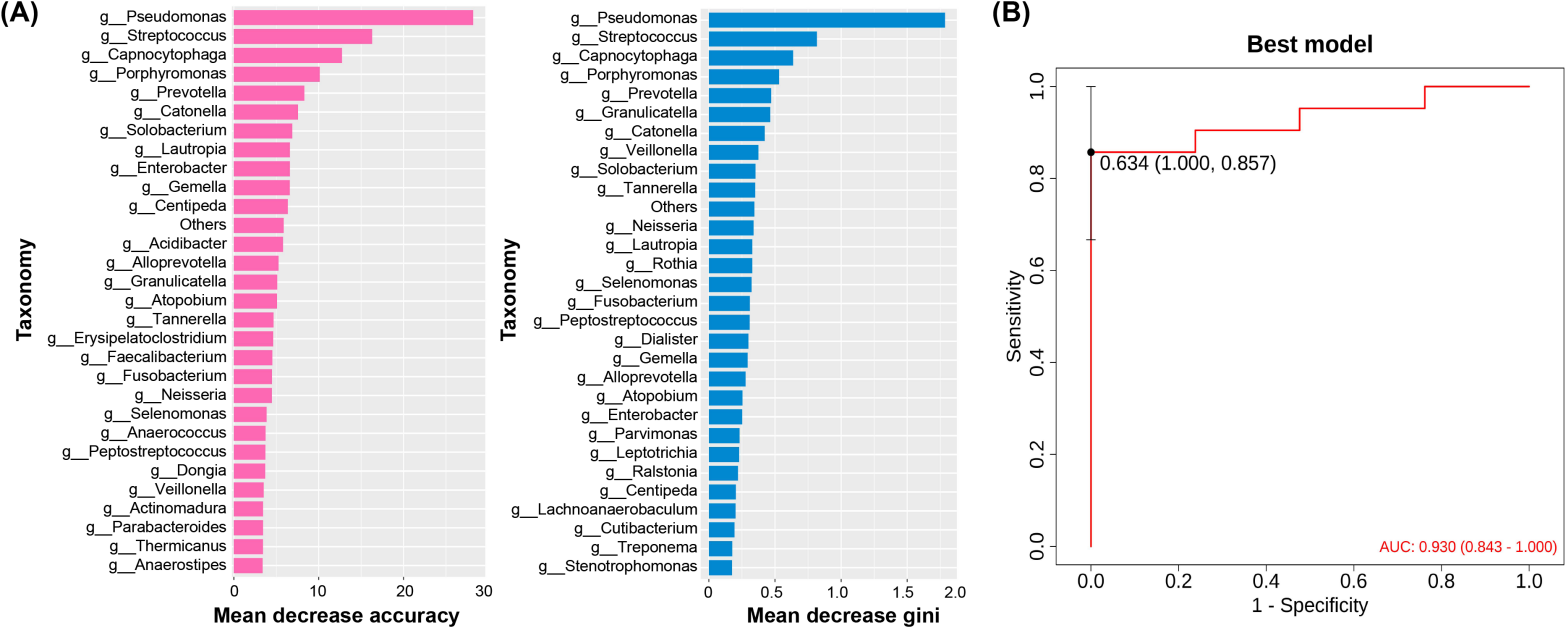
Novel microbial community identification classification model based on random forest. **(A)** The OTUs with the greatest contribution to the model are sorted by average decrease in accuracy, **(B)** The receiver operating characteristic (ROC) curve of the random forest model.

To identify potential pathogenic microbes associated with PAP patients, Random Forest model was further applied for screening. The results demonstrated that *Pseudomonas* (AUC = 0.939) and *Streptococcus* (AUC = 0.791) exhibited high accuracy scores (**Figure 4A**). The AUC (area under the curve) value is 0.930 (0.843, 1.000) by the Receiver Operating Characteristic (ROC) curve analysis (**Figure 4B**), and the optimal cut-off value of the model is 0.634, indicating the high efficacy and accuracy of the model. It is worth noting that subgroup analysis revealed that 8 of 21 patients had *Streptococcus >*1% at admission, of whom 5 also had *Pseudomonas >*10%, suggesting possible co-infection in a subset of patients (**Supplementary Figure 4**).

### The functional differences in LRT microbial communities of PAP patients

**Figure 5:**
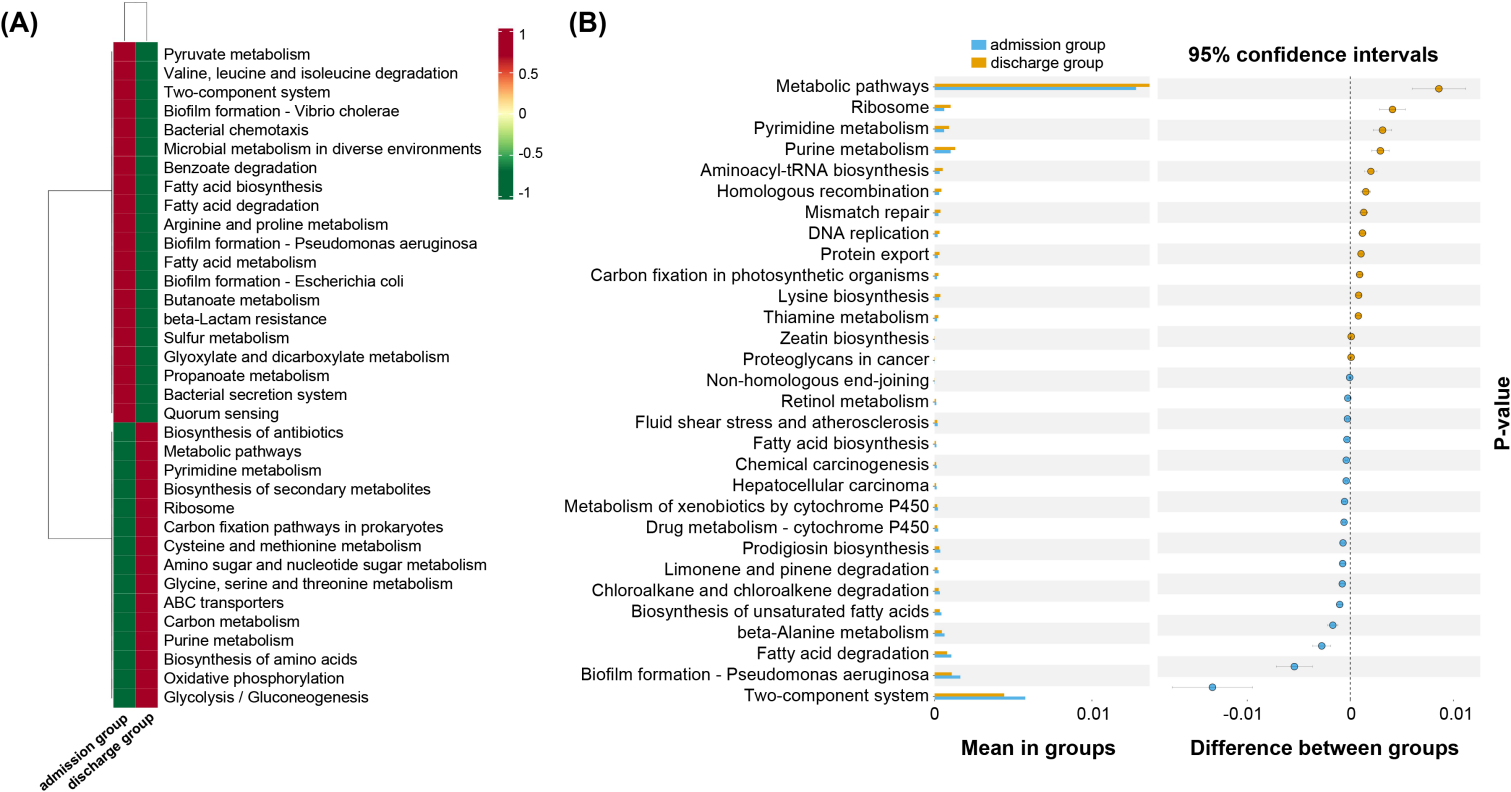
The relative abundances of level 3 functional metabolic pathways in treatment onset and day 8–10 of elderly PAP patients based on Tax4Fun2 version 1.1.6 (http://tax4fun.gobics.de/). **(A)** Function annotation clustering heat map, **(B)** T-test based on database annotation results. *p < 0.05.

To study the potential microbial interactions before and after antibiotic treatment of PAP patients, Tax4Fun2 was used to predict the functional profiles of the bacterial communities, followed by hierarchical clustering to identify functional differences between the admission and discharge groups. A total of 364 functional metabolic pathways in 6 categories were predicted (**Supplementary Figure 5**). The top 35 abundant functions across all samples were selected based on their total abundance in the database and visualized in a heatmap, followed by clustering based on functional differences (**Figure 5A**). T-tests were further performed to identify statistically significant differences in metabolic functions between groups.

Compared with the hospitalized group, the levels of pyrimidine and purine metabolism, DNA replication, lysine biosynthesis, and thiamine metabolism were significantly enhanced in the discharged group, while the levels of biofilm formation, fatty acid metabolism, alanine metabolism, and drug metabolism of cytochrome P450 were significantly decreased (*p* < 0.01) (**Figure 5B**). The findings suggest that antibiotic treatment leads to profound changes in the functional capacities of the lung microbiome in PAP patients.

### Effects of different antibiotic regimens on LRT microbial communities in PAP patients

**Figure 6:**
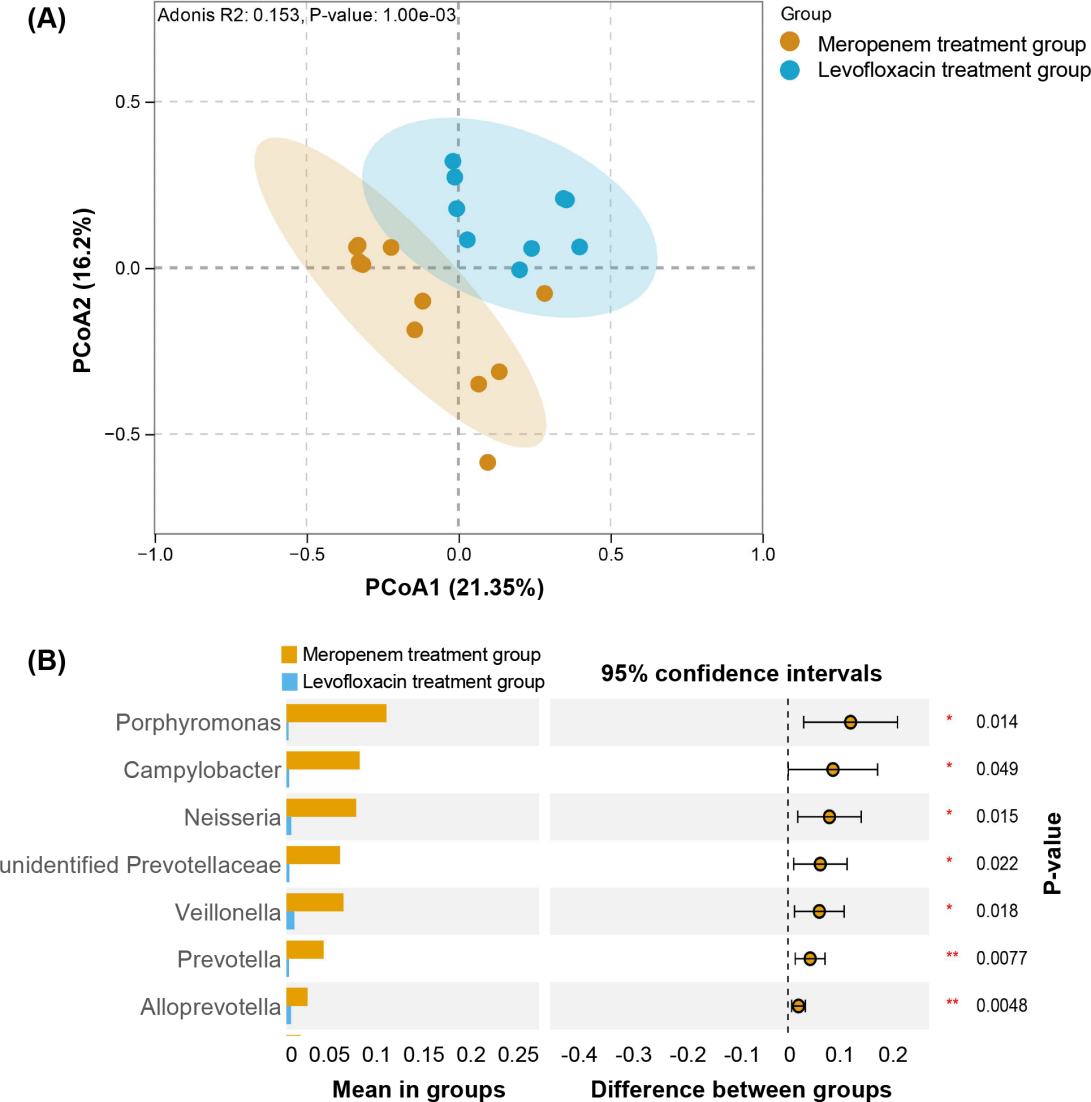
The characteristics of BALF microbiota at genus level in patients treated with different antibiotics. **(A)** PCoA of BALF microbiota in patients after treatment, **(B)** Analysis of significant differences in BALF microbiota after treatment based on T-test. Samples collected after treatment with meropenem (orange), samples collected after treatment with levofloxacin (blue). *p < 0.05.

Based on the AST of Pae isolates from BALF samples, 12 patients received meropenem-based therapy and 9 received levofloxacin-based therapy. To compare the effects of these antibiotic regimens on the LRT microbiota, we conducted PCoA analysis of the BALF microbiota at the genus level, which revealed significant differences in the lung microbiota between the levofloxacin and meropenem treatment groups (**Figure 6A**). PCoA of the BALF microbiota at the genus level was performed for patients treated with these two antibiotics, the results revealed significant differences in the lung microbiota between the levofloxacin and meropenem treatment groups (**Figure 6A**). Further analysis of species-level significant differences showed that, compared to the levofloxacin group, patients treated with meropenem exhibited significantly higher abundance of *Porphyromonas*, *Campylobacter*, *Neisseria*, and *Prevotella* in their lung microbiota, while no significant differences were observed in other species (*p* < 0.05) (**Figure 6B**).

## Discussions

Elderly individuals often suffer from a variety of underlying diseases like diabetes and hypertension, which can lead to weakened immune function (Kolahian et al., 2015; Xiao and Harrison, 2020). Moreover, the history of smoking and alcohol consumption can also lead to decreased immune system function, increasing the risk of infection (Samuelson et al., 2021; Zhang et al., 2022). This diminished immunity is thought to accelerate disease progression and enhance the susceptibility of respiratory pathogens (Langelier et al., 2018). For example, in Type 2 diabetic patients, the total antioxidant status of the lungs is significantly inhibited, leading to infiltration of mononuclear cells and edema in the trachea and lung, further exacerbating inflammation (Kolahian et al., 2015). Cunningham et al. also demonstrated an increased susceptibility to *Klebsiella pneumoniae* (Kpn) and *Streptococcus pneumoniae* (Spn) pneumonia in F1 offspring of a humanized alcohol-microbiota mouse model (Cunningham et al., 2023). Given the new therapeutic challenges posed by the steady rise of multidrug-resistant respiratory pathogens like Pae and Kpn, we are rapidly approaching the advent of a post-antibiotic era. Further research has identified additional risk factors in elderly PAP patients, including prolonged mechanical ventilation, tracheostomy, and the use of three or more antibiotics (Rocha et al., 2008).

The complexity of antibiotic selection is also exacerbated in elderly patients with PAP, who exhibit high resistance rates. Understanding the distribution of respiratory flora in this demographic is crucial for optimizing clinical treatment plans, although existing data on the microbiome diversity in elderly PAP patients remain unclear. In this study, a longitudinal analysis of patients’ hematological indicators from admission to discharge revealed significant decreases in WBC count and concentration of CRP, indicative of potential resolution in the systemic inflammatory response, which is a critical facet of disease management. Conversely, notable increases in LYM and PLT counts were observed, suggesting concurrent improvements in immune function and platelet production, likely attributable to therapeutic intervention. These findings suggest that serial assessment of WBC, CRP, LYM and PLT may provide clinically informative measures of treatment response within this cohort. However, studies incorporating hard clinical endpoints, such as intensive care unit transfer and 30-day mortality, will be required to establish their prognostic utility.

16S rRNA gene amplicon sequencing was used to characterize the lung microbiota of elderly PAP patients upon admission and discharge, followed by exploring the diversity and biomarkers of microbiota community. Alpha diversity indices revealed a significant shift in Shannon index but not in richness estimators (Chao1/ACE), suggesting antibiotic treatment primarily altered community dominance patterns rather than total species number. This aligns with Shannon’s sensitivity to low-abundance taxa redistribution. This finding is consistent with previous results that pathogen overgrowth in pneumonia patients can lead to decreased microbial diversity (Sakwinska et al., 2014).

Beta diversity analysis revealed a dramatic difference in lung microbiota community composition between admission and discharge (PCoA, *p* < 0.001), indicating that both Pae infection and antibiotic treatment collectively shape the LRT microbiota in PAP patients. Previous studies have also revealed the pulmonary environment in lower respiratory tract infections (LRTI) patients is characterized by distinct microbial shifts, which are influenced by factors such as inflammation, immune response, and antibiotic use. These environmental changes can alter the composition and diversity of the microbial communities in the lungs (Pérez-Losada et al., 2017). Furthermore, the consistency between NMDS and PCoA results suggests the robustness of the data, which suggests a common dysbiosis pattern at admission (likely due to the dominance of Pseudomonas aeruginosa) and greater diversity at discharge, potentially reflecting microbial expansion or community restructuring post-treatment.

Machine learning approach based on random forest algorithm was employed for feature selection and biomarker identification, renowned for its high accuracy and robustness (Couronné et al., 2018; Lai et al., 2021). It has previously been applied to screen new non-invasive biomarkers for colorectal cancer in oral microbiota and general-level biomarkers in gut microbiome for children with juvenile idiopathic arthritis, affirming the model’s versatility in screening for novel bacterial genera and biomarkers (Qian et al., 2020; Zhang et al., 2020). The Random Forest model showed potential for predicting treatment response, though validation in external cohort is required for clinical application. Future optimizations should aim to improve sensitivity.

To explore the potential novel biomarkers in elderly PAP patients, the random forest analysis was constructed in the present study. The obtained results showed that the random forest model performed promising discriminative capacity in identifying microbial biomarkers of PAP. While *Pseudomonas* is a known PAP pathogen, its co-occurrence with relevant biomarker (e.g., *Streptococcus*) may enhance diagnostic precision. Subgroup analysis further suggested possible polymicrobial infection in a subset of PAP patients. While these microbial signatures distinguished infected states from baseline in our cohort, validation against healthy controls is required for diagnostic utility. By accurately identifying key pathogens, clinicians can enhance diagnostic accuracy and guide targeted therapeutic interventions for improved patient outcomes. However, the cohort size (n = 21) limits generalizability, further validation studies and clinical trials in larger cohorts are warranted to corroborate these findings and assess the model’s performance across diverse patient cohorts and clinical settings.

Prior research suggests that the function of the microbiome depends on the individual and interactive effects of the environment and community structure, with key roles in maintaining immunological homeostasis in lung mucosa (Orland et al., 2019). Microbe-host and microbe-microbe interactions also play important roles on the susceptibility of pathogens by metabolites mediated immunological progresses (Wu and Segal, 2018). In the present study, functional prediction suggests that antibiotic therapy induces substantial shifts in the functional capacities of the lung microbiome in PAP patients. The observed enhancements in essential metabolic pathways, such as pyrimidine and purine metabolism DNA replication and lysine biosynthesis may signify microbial adaptation or recovery processes, potentially reflecting the restoration of physiological homeostasis within the lower respiratory tract. Conversely, the decline in pathways associated with biofilm formation and drug metabolism implies the efficacy of antibiotic intervention in mitigating microbial colonization and altering drug processing mechanisms within the lung microenvironment (Nickerson et al., 2024).

It is estimated that 65-80% of bacterial infections are involved in biofilm by protecting them from environmental factors, host antibodies, and phagocytes (Guerra et al., 2022). Pel, Psl and Alg operons present in Pae are responsible for the biosynthesis of extracellular polysaccharide, which plays a crucial role in cell-cell and cell-surface interactions during biofilm formation, thereby contributing to the complexity and life-threatening nature of *Pseudomonal* infections (Brandenburg et al., 2019). These findings echo earlier studies that the ability of biofilm formation are crucial virulence and resistance trait for pathogens, including Kpn and Pae (Lynch and Zhanel, 2022). Overall, these results underscore the dynamic nature of microbial functional dynamics in response to therapeutic interventions in PAP patients.

Levofloxacin and meropenem are both commonly used drugs for treating LRTI (Redgrave et al., 2014). In the present study, regimen assignment was based on AST results for patient-derived *P. aeruginosa* isolates rather than randomization, introducing the possibility of indication bias. Nevertheless, the differential impact of meropenem versus levofloxacin likely stems from their distinct spectra of activity and ecological effects (Bush and Bradford, 2016). Meropenem-induced eradication of *Pseudomonas* may enables the proliferation of anaerobic pathogens through niche competition (Louie et al., 2015). By contrast, levofloxacin targets both *Pseudomonas* and facultative anaerobes (e.g., *Neisseria*), creating broader suppression of potential pathogenic bacteria. Previous studies have shown that *Porphyromonas*, *Campylobacter*, *Neisseria*, and *Prevotella* are typical pathogenic bacteria that negatively affect human health (Baerentsen et al., 2022; García-Sánchez et al., 2018; Reyes, 2021; Sharma et al., 2022).

In this cohort, meropenem-based therapy was associated with a higher relative abundance of these taxa. However, culture-based validation and larger prospective studies are required to confirm this observation. When levofloxacin is unsuitable and meropenem use is undesirable, alternative antipseudomonal agents, including piperacillin-tazobactam, ceftazidime and ceftolozane-tazobactam, should be selected on the basis of AST results and current IDSA antimicrobial-resistance guidance (Tamma et al., 2025).

This study has several limitations. First, we focused on microbial diversity and biomarker identification using culture-independent technology, rather than conducting experimental studies to pinpoint the functional roles of specific species in LRTI progression. Second, because the primary objective was to characterize longitudinal changes within PAP patients from admission to discharge, differences between these two time points probably reflect the combined effects of ongoing infection and antibiotic exposure. Future studies should therefore include age-matched controls to define baseline LRT microbiota composition and strengthen interpretation of the findings. Because BALF collection from healthy older adults is ethically and practically challenging, lower-risk sampling strategies, such as induced sputum collection, may provide a feasible alternative. Third, the timing and sample size of this study were limited, therefore, co-occurrence network analysis was not performed. Future research with larger and more comprehensive sampling should investigate microbial competition, cooperation, and niche partitioning in the LRT of PAP patients, as well as their relationships with clinical phenotypes. Finally, meta-analyses of publicly available BALF 16S rRNA datasets across different geographic settings may help determine whether regional or demographic factors shape LRT microbiota composition, and this remains a priority for future work.

## Conclusions

In conclusion, our study elucidates the critical role of respiratory tract microbiome dysbiosis in the pathogenesis of senile PAP. This provides novel insights into the microbiome’s composition and function in both physiological and pathological states. Additionally, we identified Pae, Spn and Pme as potential biomarkers to predict the risk of LRTI progress, enhancing the diagnostic and management strategies for elderly PAP patients. Moving forward, the developing innovative strategies for diet, as well as for regulating the microbiome will be imperative for both preventive and therapeutic interventions against respiratory infections.

## Data Availability

The datasets presented in this study can be found in online repositories. The names of the repository/repositories and accession number(s) can be found below: https://www.ncbi.nlm.nih.gov/, PRJNA1103868.
