# Supplementary material for: Lower respiratory tract microbiota characteristics in patients with Pseudomonas aeruginosa pneumonia during antibiotic therapy
Source: Front Cell Infect Microbiol. 2026 May 13;16:1777821. doi: 10.3389/fcimb.2026.1777821 (PMC13212292; doi:10.3389/fcimb.2026.1777821)
Supplement: Supplementary file 1 [file DataSheet1.pdf]

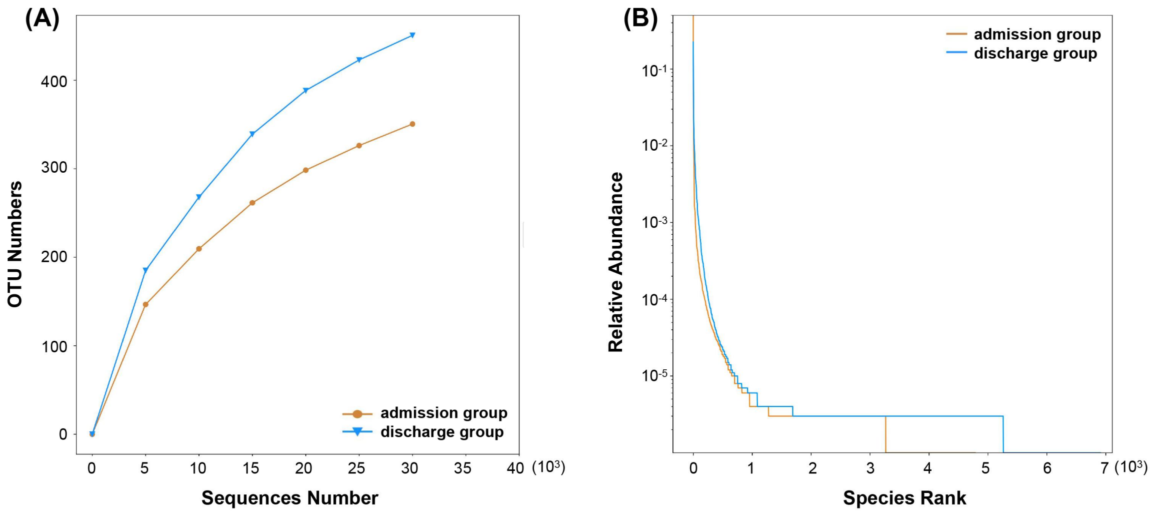

Figure S1. The dilution curves (A) and rank abundance curves (B) of 42 samples by two groups.

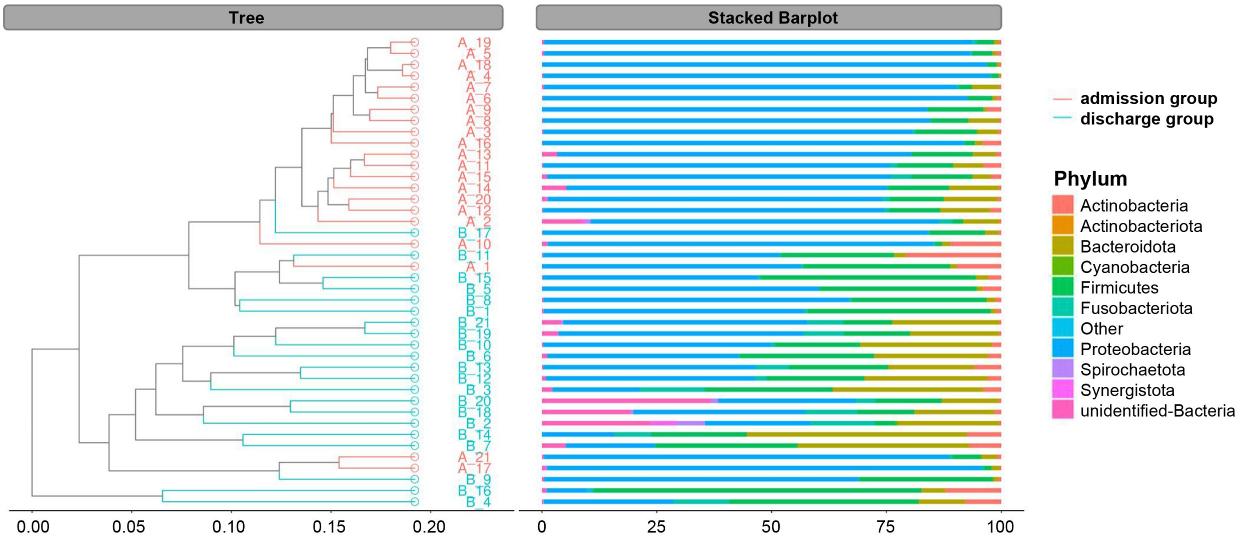

Figure S2. UPGMA clustering tree based on species Weighted Unifrac distance.

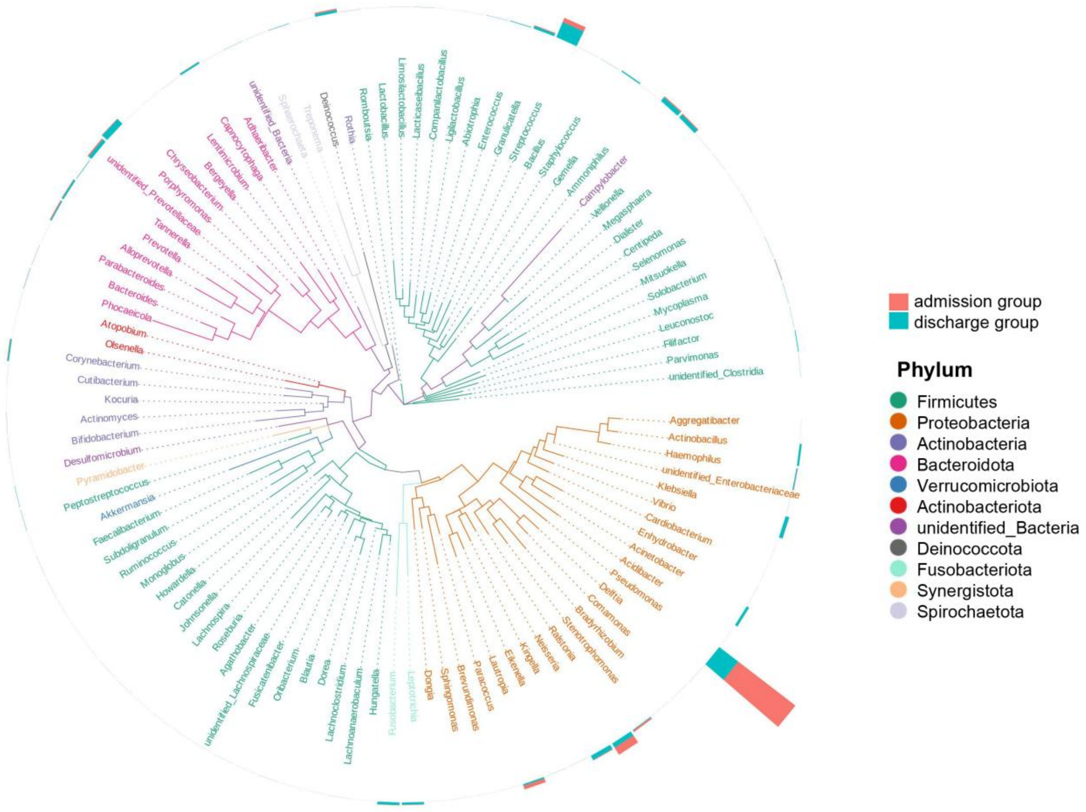

Figure S3. Phylogenetic tree of the top 100 generic-level species.

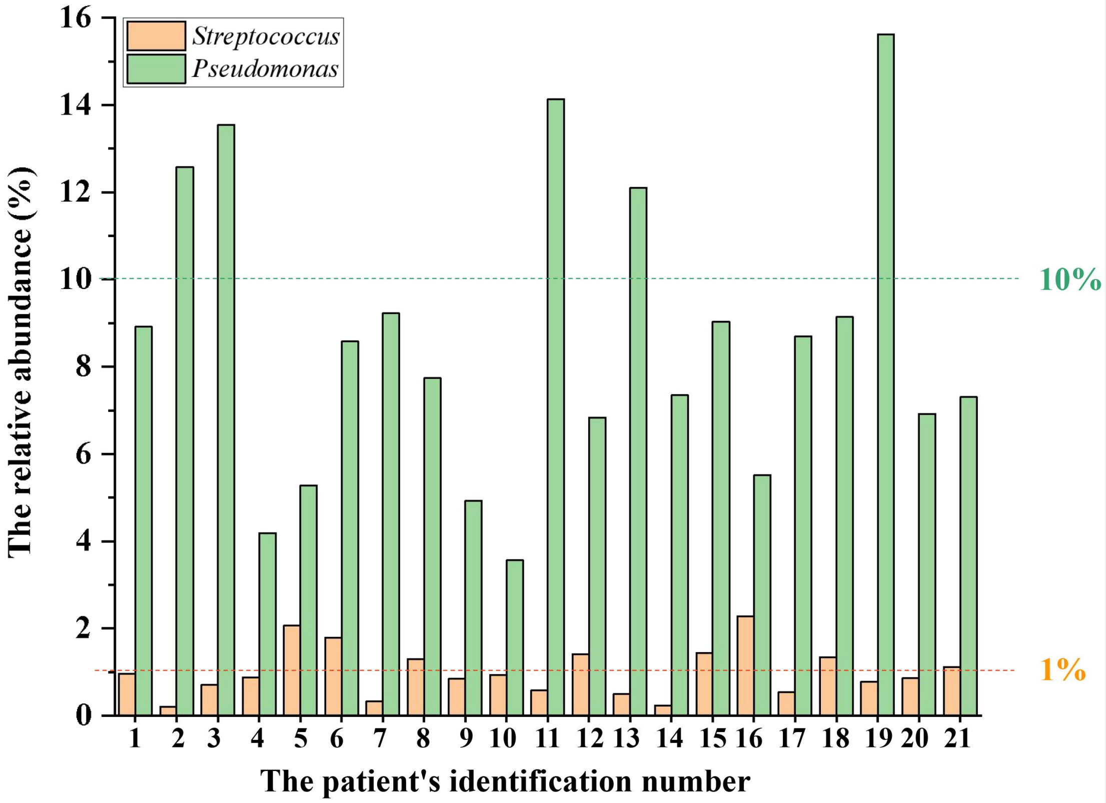

Figure S4. The relative abundance distribution of *Streptococcus* and *Pseudomonas* in 21 patients at the time of admission.

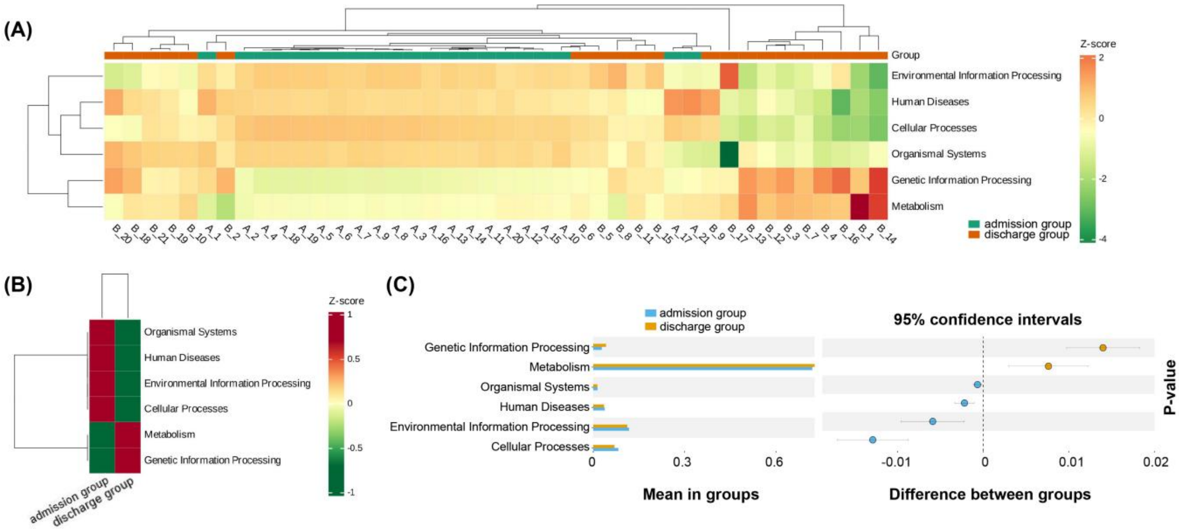

Figure S5. The relative abundances of level 1 functional metabolic pathways in treatment onset and day 8-10 of elderly PAP patients based on Tax4Fun2 version 1.1.6 (http://tax4fun.gobics.de/). (A) Functional annotation clustering heat map of each sample; (B) Function annotation clustering heat map; (C) T-test variance analysis based on database annotation results. *$p < 0.05$.
