# Supplementary material for: Lower respiratory tract microbiota characteristics in patients with Pseudomonas aeruginosa pneumonia during antibiotic therapy
Source: Front Cell Infect Microbiol. 2026 May 13;16:1777821. doi: 10.3389/fcimb.2026.1777821 (PMC13212292; doi:10.3389/fcimb.2026.1777821)
Supplement: Supplementary file 2 [file DataSheet2.pdf]

Table S1 The comprehensive quality assessment of sequencing data for admission (A) and discharge groups (B).

Sample name	Raw reads	Clean reads	Effective ratio(%)	Mean_length	Q20(%)	Q30(%)	GC(%)
A_1	112,793	108,018	73.9	427	96.2	90.09	52.79
A_2	117,868	110,429	83.8	426	95.46	88.81	51.41
A_3	118,937	113,373	81.8	427	95.86	89.51	51.92
A_4	113,476	107,355	92.02	429	95.55	88.89	51.73
A_5	111,530	106,219	84.76	428	95.89	89.58	52.06
A_6	111,018	105,254	87.27	428	95.63	89.06	52.01
A_7	119,735	113,851	83.58	428	95.59	88.97	51.69
A_8	113,685	107,958	85.57	428	95.67	89.11	51.71
A_9	111,099	104,873	83.01	428	95.48	88.74	52.12
A_10	111,881	106,997	80.27	427	96.31	90.37	53.21
A_11	116,843	111,241	74.12	428	95.93	89.56	52.04
A_12	111,128	105,092	75.76	428	95.69	89.16	51.97
A_13	111,589	105,469	83.65	427	95.71	89.22	51.66
A_14	111,133	105,316	79.64	427	95.86	89.52	51.52
A_15	111,444	106,464	74.43	427	95.99	89.75	51.97
A_16	115,188	108,957	83.9	428	95.74	89.3	52.57
A_17	112,225	107,326	86.57	428	96.25	90.18	52.86
A_18	112,638	106,575	89.57	429	95.59	89.03	51.83
A_19	111,255	105,838	81.25	428	95.84	89.52	52.22
A_20	111,310	105,137	80.78	427	95.54	88.88	51.76
A_21	100,551	96,979	82.58	428	96.5	90.69	52.73
B_1	112,059	108,184	71.3	429	96.51	90.64	51.62
B_2	111,095	106,736	78.86	417	96.54	90.58	50.35
B_3	113,262	107,260	64.69	423	96.2	90.01	51.21
B_4	111,684	107,834	81.73	425	96.61	90.87	52.2
B_5	111,407	105,986	80.68	428	95.89	89.49	52.16
B_6	117,060	112,305	75.42	427	96.14	89.92	51.71
B_7	115,612	111,332	75.85	425	96.39	90.33	51.73
B_8	111,304	106,986	74.64	428	96.23	90.12	51.94
B_9	105,178	102,147	85.43	429	96.8	91.18	52.71
B_10	112,426	107,181	75.36	427	96.22	90.12	51.81
B_11	111,569	105,839	78.66	425	95.86	89.41	52.7
B_12	111,526	107,427	65.47	426	96.6	90.82	51.69
B_13	119,521	114,730	69.07	425	96.49	90.62	52.02
B_14	113,332	109,826	73.43	424	96.54	90.51	51.58
B_15	113,213	107,588	80.87	428	95.8	89.4	51.77
B_16	112,720	106,467	85.98	426	95.74	89.08	52.23
B_17	111,578	107,300	77.07	429	96.46	90.5	54.6
B_18	113,120	108,256	65.59	420	96.33	90.32	50.9
B_19	112,406	107,796	68.03	425	96.23	90.2	51.2

B_20	111,533	107,242	74.67	417	96.6	90.9	50.55
B_21	113,034	107,824	68.97	425	96.1	89.94	51.26
